# Cold agglutinin-induced hemolytic anemia during room temperature fluid resuscitation: a case report

**DOI:** 10.1186/s13256-021-02784-8

**Published:** 2021-04-16

**Authors:** Yosuke Kawai, Miyoshi Deguchi, Naoko Mizouchi, Satoru Yoshida, Ken Kumagai, Yasuo Hirose

**Affiliations:** Niigata City General Hospital Emergency & Critical Care Medicine, 463-7 Shumoku, Chuo-ku, Niigata, 950-1197 Japan

**Keywords:** Cold agglutinin disease, Hemolytic anemia, Fluid resuscitation, Diabetic ketoacidosis, Intensive care

## Abstract

**Background:**

Cold agglutinin disease can cause the agglutination of red blood cells and hemolytic anemia due to cold temperature. Herein, we report a case of progressive hemolytic anemia due to cold agglutinin disease during fluid resuscitation and in the absence of exposure to cold.

**Case presentation:**

A 71-year-old Japanese man was admitted to the emergency department with signs of hypotension and disturbed consciousness. He was diagnosed with diabetic ketoacidosis, and treatment with fluid resuscitation and insulin infusion was initiated. Laboratory test results obtained the following day indicated hemolytic anemia. On day 5 after admission, red blood cell agglutination was detected, and the patient was diagnosed with cold agglutinin disease.

**Conclusions:**

Cold agglutinin disease should be considered in the differential diagnosis of progressive hemolytic anemia during fluid resuscitation, even if the solution is at room temperature.

## Background

Anemia in patients admitted to intensive care is a common and significant problem, with 90% of patients developing anemia by the third day after admission, and 97% by the eighth day [1]. The most common causes of anemia in intensive care unit (ICU) patients are chronic diseases (32.4%) and blood loss (11.8%), with hemolysis being the cause in only 2.9% of cases [2].

Cold agglutinin disease (CAD) can cause red blood cell (RBC) agglutination and extravascular hemolysis in patients exposed to cold temperatures, resulting in anemia. CAD is caused by high levels of circulating cold agglutinins (CAs). To avoid exposure to cold temperatures, maintenance of room temperature, protection of the extremities and acral areas of the face with warm clothing, and avoidance of cold food or liquid consumption are recommended. Cold intravenous solutions and transfusions should be avoided in hospitalized patients.

In this study, we present a case of a patient with diabetic ketoacidosis (DKA) and progressive anemia due to CAD during fluid resuscitation, despite the intravenous infusion being administered using a solution at room temperature.

## Case presentation

A 71-year-old Japanese man was admitted to our hospital with signs of hypotension and disturbed consciousness. The patient had a history of type 2 diabetes, but due to treatment default he was not receiving any regular medication. There was no other specific medical, social, family, or surgical history. On examination, the patient appeared pale and ill, and he had livedo reticularis. The patient’s blood pressure was 85/47 mmHg, pulse rate was 125 bpm, respiratory rate was 20 breaths/min with O_2_ saturation of 99% in room air, and rectal temperature was 33 ℃. The neck was supple. Results of the cardiovascular examination were normal, lungs were clear to auscultation, and the results of the abdominal examination were unremarkable. On neurological examination, his pupils were dilated to approximately 5 mm and were nonreactive to light; the Glasgow Coma Scale score was 6 (E4V1M1). Results from the initial laboratory tests revealed signs of macrocytic anemia (hemoglobin level 9.1 g/L, mean cell volume 112 fL), increased number of white blood cells (25,800 cells/μL), and elevated C-reactive protein level (40.4 mg/L). The patient had hyperglycemia (710 mg/dL). The arterial blood gas test showed metabolic acidosis (pH 6.723, base excess − 32.1 mmol/L) with an anion gap of 30.8. The urine test showed the presence of acetone bodies. Computed tomography without contrast of the whole body (head, chest-pelvis) showed bilateral pulmonary consolidation and mild pancreatic enlargement.

Based on hyperglycemia, presence of urinary acetone bodies, and metabolic acidosis with increased anion gap, the patient was diagnosed with DKA (Table 1). Initial treatment with fluid resuscitation (2000 mL per 1.5 h, lactated Ringer’s solution), insulin intravenous bolus of 5 U followed by intravenous continuous infusion (3 U/h), and active external rewarming with warm blankets was initiated in the emergency department. After the initial treatment, the patient was admitted to the ICU. Despite fluid resuscitation (400 mL/h), hypotension persisted, and treatment with norepinephrine and vasopressin was initiated. Twelve hours after admission to the ICU, his glucose level had decreased to 385 mg/dL, and 7600 mL of crystalloid fluid was administered. Complete blood count showed the progression of anemia. Biochemical analysis revealed elevated bilirubin levels, and he received a non-warmed blood transfusion. On day 3, his hemodynamic parameters improved. Thus, therapy with norepinephrine and vasopressin was discontinued, and the infusion rate was reduced (Table 2).

**Table 1:** Laboratory data on admission

Laboratory parameters	Patient’s values	Reference range
White blood cell (cells/μL)	25,800	3300–8600
Hemoglobin (g/dL)	9.1	13.7–16.8
MCV (fL)	112	83.6–98.2
Platelets (cells/μL)	291,000	158,000–340,000
Sodium (mmol/L)	135	138–145
Potassium (mmol/L)	5.1	3.6–4.8
Chloride (mmol/L)	102	101–108
Urea nitrogen (mg/dL)	60.2	8–20
Creatinine (mg/dL)	1.48	0.65–1.07
C-reactive protein (mg/dL)	4.42	<0.14
AST (IU/L)	55	13–30
ALT (IU/L)	30	10–42
LDH (IU/L)	526	124–222
Total bilirubin (mg/dL)	1.2	0.4–1.5
Glucose (mg/dL)	710	73–109
ABG: pH	6.723	7.35–7.45
ABG: bicarbonate (mmol/L)	2.2	21–27
Urine acetone body	3+	

*MCV* Mean corpuscular volume, *AST* aspartate aminotransferase, *ALT* alanine aminotransferase, *LDH* lactate dehydrogenase, *ABG* arterial blood gas

**Table 2:** Laboratory findings during patient’s stay in the intensive care unit

Laboratory parameters	Days after admission to intensive care unit				
	1	2	3	4	5
Hemoglobin (g/dL)	9.1	6.1	8.5^a^	9.3	10.4
Total bilirubin (mg/dL)	1.2	5.0	1.1	1.8	1.5
Direct bilirubin (mg/dL)		3.4	0.5	0.5	
Infusion rate (mL/h)	1500 → 400	400 → 160	160 → 20	20	20

Laboratory findings revealed progression of anemia with an elevation in total bilirubin levels accompanying a high infusion rate

^a^The patient received a transfusion of 560 mL of red blood cells on day 2

**Fig. 1:**
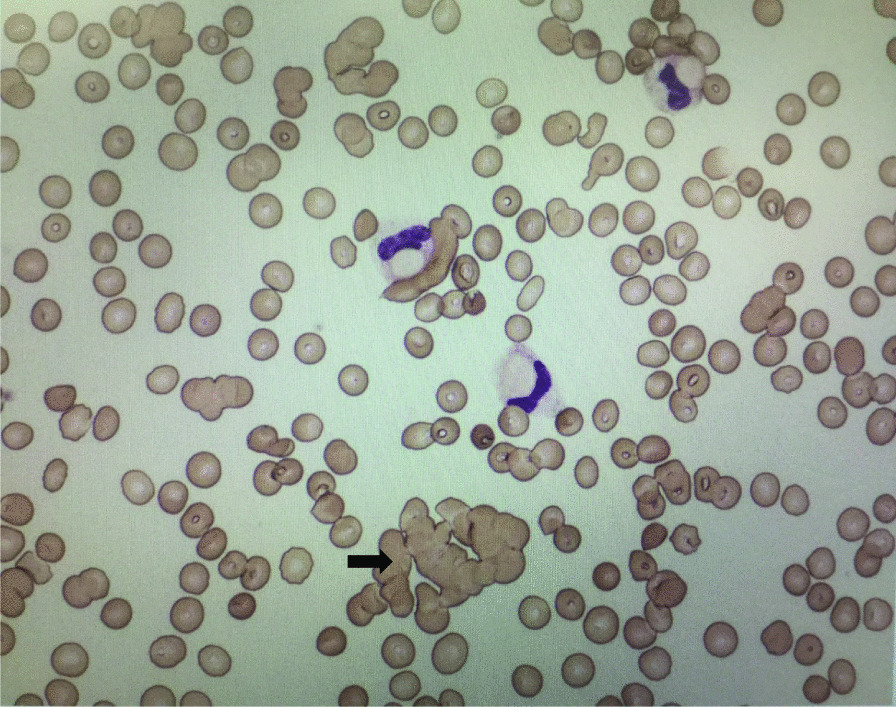
Peripheral blood smear showing red blood cell agglutination (arrow)

On day 5 of the patient’s ICU stay, RBC agglutination was observed (Fig. 1). Based on RBC agglutination and elevated bilirubin levels, CAD was suspected. The result of the direct Coombs test was positive, and the CA titer test revealed an elevated autoantibody titer (1:8192 at 4 ℃). The patient was therefore diagnosed with CAD. No bacterial or viral infections causing CAD or DKA were detected (Table 3). He was discharged from the ICU on day 6. As a preventive measure, the patient was recommended to avoid cold exposure. During the hospital stay, there was no recurrence of anemia. On day 32 following presentation, he was discharged from the hospital.

**Table 3:** Results of laboratory tests performed to diagnose cold agglutinin disease and determine background infections

Laboratory tests	Patient’s results/values	Reference range
Direct Coombs test	Positive	
Indirect Coombs test	Negative	
CA titer at 4 ℃	1:8192	1:< 256
EBV VCA IgM	< 10	< 10
EBV VCA IgG	160	< 10
EBNA	20	< 10
CMV antigenemia	Negative	
CMV IgM	Negative	
CMV IgG	Negative	
*Mycoplasma pneumoniae* PA titer	< 40	< 40
*Mycoplasma pneumoniae* LAMP	Negative	

*EBV VCA* Epstein–Barr virus viral capsid antigen, *EBNA* EBV nuclear antigen, *CMV* Cytomegalovirus,* Ig* immunoglobulin, *PA* particle agglutination, *LAMP* loop-mediated isothermal amplification

## Discussion

A case of cold agglutinin disease is extremely uncommon in the emergency department. As our patient was not exposed to cold temperatures in the ICU, there was no suspicion of CAD at presentation. We therefore decided to investigate the mechanism of CAD hemolytic anemia during room temperature fluid resuscitation.

Previous studies have demonstrated that CAs bind to antigens on the surface of RBCs in the acral areas where the blood temperature is lower than the body temperature and that they recruit components of the complement pathways. Upon warming of the central circulation to body temperature, CAs detach from the RBCs, but C3b remains bound. Some of the C3b-coated RBCs are trapped by macrophages mainly in the liver, resulting in extravascular hemolysis [3].

According to the pathophysiology described above, we considered two possible mechanisms of hemolytic anemia in this case. The first is that the rapid room temperature infusion decreased peripheral intravascular temperature, resulting in the activation of CAs. Although CAs usually agglutinate RBCs at 4 °C, most clinically important CAs act at temperatures exceeding 28–30 °C [4]. A previous study showed that during a 30-min infusion of 30 mL/kg room temperature saline solution into healthy subjects, the core temperature decreased by 0.5 °C [5]. In light of this study, it is reasonable to suggest that rapid intravenous room temperature infusion also reduces peripheral intravascular temperature.

The second possible mechanism is that rewarming and subsequent improvement in hemodynamics by fluid resuscitation affect the delayed identification of hemolytic anemia. On admission, the patient had livedo reticularis, which is a manifestation of blood flow disturbance and vessel dilation. In CAD patients, agglutinated RBCs in the cooler peripheral areas cause a hyperviscosity of blood flow and blood stasis in the capillary venules, which in turn leads to livedo reticularis [6]. There is a possibility that our patient was exposed to cold temperatures before hospitalization. As active external rewarming was performed in the hospital, CAs then detached from RBCs, and capillary stasis was eliminated; at the same time, fluid resuscitation increased blood return to the central circulation, resulting in hemolysis of C3b-coated RBCs in the liver.

There are some reports of incidental diagnosis of CAD in medical practice where the clinical symptoms presented intraoperatively during hypothermic cardiopulmonary bypass [7, 8]. Barbara et al. recommend that patients with a history or evidence of hemolysis and agglutinations should undergo CA and thermal amplitude tests in conjunction with a hematologic consultation before cardiac surgery [9].

In addition, CAD should be a differential diagnosis of progressive hemolytic anemia during rapid intravenous infusion, even if the solution used is at room temperature. In emergency care, fluid resuscitation is often administered. In patients with hemolytic anemia, peripheral blood smear, Coombs test, and CA test should be performed, and infusion or transfusion should be changed to a warmed one.

## Conclusions

In this report, we present a case of hemolytic anemia during fluid resuscitation of a patient with DKA. CAD should be considered as a differential diagnosis in patients developing hemolytic anemia during fluid resuscitation, even if the solution used for resuscitation is at room temperature.

## Data Availability

Not applicable.

## Abbreviations

CAD: Cold agglutinin disease
CA: Cold agglutinin
DKA: Diabetic ketoacidosis
ICU: Intensive care unit
RBCs: Red blood cells
